# Next-generation sequencing in the etiological diagnosis of severe childhood pneumonia

**DOI:** 10.3389/fped.2026.1864412

**Published:** 2026-09-01

**Authors:** Xiao Liu, Rongjie Chen, YinJie Li, Dengran Li, Zhe Xu

**Affiliations:** 1 Guangyuan Central Hospital Affiliated to North Sichuan Medical College, Nanchong, Sichuan, China; 2 Department of Pediatrics, Guangyuan Central Hospital, Guangyuan, Sichuan, China

**Keywords:** bronchoalveolar lavage fluid, metagenomics, next-generation sequencing, pathogen diagnosis, pathogen spectrum, precision medicine, severe pneumonia in children

## Abstract

Pneumonia is the leading cause of death for children, particularly those aged 3–5 years old. The majority of the 740,000 annual global deaths of children under five years old occurred in developing countries in 2019. Severe childhood pneumonia (SCAP) causes death and disability in children, as it may trigger pleural effusion, respiratory failure, bacteremia and multiple organ failure. Traditional pathogen detection techniques including microscopy, culture and polymerase chain reaction (PCR) feature narrow detection coverage, lengthy detection cycles and low sensitivity. These defects frequently lead to undetermined infectious etiology, forcing clinicians to administer empirical broad-spectrum antibiotics. Metagenomic next-generation sequencing (mNGS) has emerged as an innovative, high-throughput, untargeted detection technology in recent years, which greatly promotes the etiological diagnosis of infectious diseases. Published meta-analyses show that the pathogen detection rate of mNGS using bronchoalveolar lavage fluid (BALF) samples collected from children can reach 85.83%, which is markedly higher than the 49.97% detection rate of conventional testing approaches. This review systematically elaborates how mNGS reconstructs the cognition of pathogen spectrum in children with severe pneumonia: this technology accurately identifies complex mixed infection patterns, detects pathogens that cannot be captured by traditional testing, and redefines the clinical boundary between microbial colonization and invasive infection. Combined with latest clinical research data, this paper analyzes the core value of mNGS in optimizing treatment schemes, reducing irrational antibiotic use and realizing precision therapy for severe pneumonia in children. Meanwhile, the current limitations and future development directions of this technology are discussed.

## 1 Introduction: from blind empirical diagnosis to full-Spectrum identification — a paradigm shift in pathogen detection for severe pneumonia in children

According to the 2021 Global Burden of Disease Study (1), lower respiratory tract infections have long been one of the top diseases contributing to global disease burden and a major lethal factor for children under five years old. Pneumonia ranks first among all death causes of children under five worldwide. In 2019, pneumonia caused approximately 740,000 deaths of children in this age group globally, among which 181,000 deaths happened in China, accounting for 22% of all deaths of children aged 1–5 years old (2, 11, 12).

Severe pneumonia is one of the most common and intractable diseases treated in pediatric intensive care units (PICUs) (3, 13). Improper early antibiotic intervention and inappropriate treatment regimens for lung infections are the primary drivers of increased complications and mortality (4). This disease progresses rapidly, and its overall mortality remains high (14). Although antimicrobial drugs and vaccines have achieved remarkable progress in the past decades, bacterial resistance remains a key factor leading to high incidence and mortality of lower respiratory tract infections in children (1, 5, 15). Such conditions not only impose huge pressure on the public health system, but also bring heavy economic and mental burdens to children's families.

Rapid and accurate identification of pathogenic microorganisms is the prerequisite for optimizing treatment strategies and improving prognosis, yet conventional testing methods fail to confirm causative pathogens in more than half of children with pneumonia (6, 7).

Pathogen culture is recognized as the diagnostic gold standard, but it requires long incubation time: bacteria take 2–5 days, while fungi and mycobacteria need several weeks. Moreover, this method easily misses atypical and fastidious pathogens such as *Haemophilus influenzae*. Previous antibiotic exposure will inhibit the growth of pathogens and reduce culture positivity, further prolonging hospital stay (16, 17). Sputum collection also faces practical obstacles: young children cannot actively produce qualified sputum samples, and collected sputum is easily contaminated by commensal bacteria colonizing the oropharynx, which distorts test accuracy (18).

Direct microscopic smear detection is fast and low-cost, but its sensitivity and specificity are extremely poor. Test results are greatly affected by pathogen load in specimens and the professional experience of laboratory operators. Targeted PCR assays also have prominent drawbacks. They can only detect preset pathogens and cannot effectively distinguish colonization from true infection. Inconsistencies between genetic resistance markers and phenotypic drug susceptibility results may interfere with clinical judgment. PCR antigen testing achieves rapid detection with good sensitivity, but it only covers limited target pathogens. It cannot detect rare pathogens, emerging pathogens or clinically common mixed infections, so it cannot support precise clinical diagnosis (17, 20, 21).

Approximately 50% of community-acquired pneumonia cases cannot clarify pathogens through smear, culture and routine PCR, because these conventional techniques fail to identify rare and fastidious bacteria (20, 22, 23). This diagnostic deficiency forms a vicious clinical cycle: undetermined pathogen → overuse of broad-spectrum antibiotics → poor clinical prognosis. Restricted by inherent technical defects, more than half of children with severe pneumonia cannot obtain clear etiological evidence, so clinical treatment mainly relies on empirical medication. Such a model may delay disease control and aggravate the prevalence of antibiotic resistance (9). Therefore, it is urgent to develop alternative diagnostic technologies with high sensitivity and specificity (8).

To intuitively reflect the advantages of mNGS over traditional detection methods, a comparative table is listed below Table 1.

**Table 1:** Comparison of mNGS with conventional pathogen detection methods.

Detection method	Detection range	Sensitivity	Turnaround time	Workflow	Cost	Clinical advantages	Clinical limitations
Traditional Culture	Bacteria/fungi	Low	2–7 days	Simple	Low	Gold standard for drug susceptibility testing	Misses fastidious/non-culturable pathogens
Microscopy	Partial pathogens	Very low	Minutes	Simple	Low	Rapid preliminary screening	Low specificity; results heavily operator-dependent
Targeted PCR	Predefined target pathogens	Medium–High	4–8 hours	Simple	Medium	Rapid turnaround, high analytical specificity	Cannot detect unknown, rare, or co-infecting pathogens
mNGS	All microbial taxa (unbiased pan-pathogen screening)	High	12–48 hours	Complex	High	Full-spectrum identification; detects mixed infections and antimicrobial resistance (AMR) genes	High testing cost; requires rigorous laboratory quality control

mNGS, metagenomic next-generation sequencing; PCR, polymerase chain reaction; AMR, antimicrobial resistance; BALF, bronchoalveolar lavage fluid; tNGS, targeted next-generation sequencing.

All microbial detection metrics summarized from published pediatric pneumonia diagnostic meta-analyses (10, 24).

As shown in Table 1, mNGS possesses prominent strengths in detection coverage, sensitivity and identification of mixed/rare pathogens, which qualifies it as a core diagnostic tool for etiological confirmation of severe pneumonia in children.

Different from conventional testing, mNGS does not require prior prediction of potential pathogens. Untargeted mNGS adopts shotgun sequencing to perform large-scale random analysis on clinical samples or pure microbial cultures; in contrast, targeted sequencing relies on primer extension, probe capture or single/multiplex PCR amplification (25). As an innovative pathogen identification platform, mNGS can simultaneously detect bacteria, viruses, fungi and parasites in a single test (26).

Benefiting from technical breakthroughs in removing host DNA interference and cost control, untargeted whole-nucleic acid sequencing of mNGS can provide comprehensive microbial information within 12–24 h, breaking the limitations of fixed detection targets and low sensitivity of traditional assays (27). This unbiased testing mode shows unique advantages in identifying novel and rare infectious pathogens, as it does not rely on preset pathogen lists and can capture multiple microbial types synchronously (25).

A systematic meta-analysis confirmed that mNGS can lift the pathogen detection rate up to 85.83%, nearly twice that of conventional testing (OR = 3.99), providing high-level evidence to resolve clinical diagnostic difficulties (10). Multiple independent clinical studies have verified the superior diagnostic performance of mNGS for pulmonary infections in children. Zheng Yihui et al. (22) conducted a controlled trial and found that mNGS exhibits higher sensitivity than traditional culture; it can identify pathogenic microbes even when culture results are negative and is less affected by confounding clinical factors.

mNGS enables rapid and accurate pathogen screening, which supports clinicians to rationally adjust antibiotic schemes, so it is recommended as a reliable diagnostic tool for children's respiratory infections (29–31). A large number of studies have confirmed its excellent performance in identifying mixed viral infections among critically ill children with bronchopneumonia admitted to PICUs. Mixed infections are extremely common in children's pneumonia clinics, and mNGS can comprehensively detect multiple co-infecting pathogens at an early stage, providing key etiological evidence for combined targeted therapy (28, 32, 33).

For children with severe *Mycoplasma pneumoniae* pneumonia, BALF-mNGS testing shows particularly prominent advantages: its detection rate of mixed infections is far higher than traditional assays. Besides, mNGS supports early viral genotyping and viral load quantification, which is vital for evaluating the severity of critical illness and optimizing individualized treatment (23, 32). Multiple studies have verified the clinical value of BALF specimens in mNGS detection, proving that this combined testing mode can significantly improve the efficiency of etiological diagnosis for pulmonary infections and provide solid support for clinical decision-making (14, 30). In summary, BALF-based mNGS testing overcomes multiple defects of traditional diagnostic techniques, forming a fast, accurate and comprehensive new pathogen detection system for children's lung infections with great clinical promotion value.

## 2 Technical advantages and standardized operation of mNGS: breaking the diagnostic blind zone of pathogens

Relying on unique technical characteristics, mNGS provides a new solution to tackle the bottlenecks of pathogen identification, yet its clinical application demands strict multi-link quality control. At present, most clinical laboratories can complete mNGS reporting within 24–48 h, which is far faster than microbial culture (34, 35). This technology occupies an irreplaceable position in the current infectious disease diagnostic system.

The accuracy of mNGS results depends on standardized quality control covering all interconnected experimental links. In terms of sample selection, BALF is recognized as the optimal specimen for diagnosing lower respiratory tract infections, because it is directly collected from lesion sites and reduces contamination interference from upper respiratory commensal flora (26, 27, 34).

Nevertheless, clinicians need to comprehensively evaluate the clinical indication, safety and necessity of bronchoalveolar lavage (BAL) for critically ill children with severe pneumonia. BAL is clearly indicated for children with rapidly progressive pneumonia, poor response to empirical antimicrobial therapy or unknown etiology. When operated by experienced pediatric bronchoscopists following standardized procedures, BAL brings low complication rates and good tolerance among critically ill children.

BALF can truly reflect the pathogen spectrum of lesion tissues and avoid upper respiratory flora interference, so it is irreplaceable for accurate etiological diagnosis of lower respiratory tract infections. The combination of BAL sampling and mNGS brings multiple clinical benefits: early and precise pathogen confirmation, timely adjustment of antimicrobial regimens, reduction of unnecessary antibiotic use, shortened hospital stay and improved overall prognosis. Strict risk control and standardized operation specifications are required to prevent intraoperative and postoperative complications, including preoperative assessment of respiratory and hemodynamic status, complete aseptic manipulation and continuous vital sign monitoring during and after lavage.

Pretreatment for removing host cells and free human nucleic acid is a key step to improve detection sensitivity. Subsequent experimental procedures include nucleic acid extraction, library construction and high-throughput sequencing to generate massive sequencing reads. Bioinformatic analysis serves as an indispensable core link: technicians first filter more than 99% of host human sequences, then align remaining reads with professional microbial databases to complete species identification.

This analytical process faces multiple challenges, including uneven database quality, difficulty distinguishing true pathogens from contaminating microbes, and balancing false-positive and false-negative results. Uniform stringent standards and complete quality control systems are required to solve these problems (36).

Multiple clinical studies have confirmed the superior diagnostic performance of mNGS. A retrospective study of 127 children with severe or refractory pneumonia showed that the overall pathogen detection rate of bronchoalveolar lavage fluid metagenomic next-generation sequencing (BALF-mNGS) was as high as 96.06%, significantly higher than the 72.44% detected by conventional microbiological tests (*P* < 0.001). This result confirms that this technique has superior ability in identifying pathogenic microorganisms, particularly in detecting bacteria and fungi (53).

Jiang L et al. (24) conducted a controlled study and found that the pathogen detection rate of BALF-mNGS reached 84%, while traditional culture only achieved 48%. Meanwhile, the detection positive rate of BALF specimens (82.14%) was significantly higher than blood samples collected from the same group of children (48.21%). Wang J et al. (32) reported that the sensitivity of mNGS was 97.2%, greatly exceeding the 13.9% of conventional testing (*P* < 0.01), while its specificity (63.2%) was slightly lower than traditional methods (94.7%, *P* = 0.07). This result reminds clinicians to carefully screen false-positive signals caused by colonizing flora during result interpretation.

mNGS has unique advantages in identifying anaerobic bacteria, fungi and various mixed infections (9, 37). A cohort study focusing on children with refractory pneumonia showed that the diagnostic sensitivity of mNGS was as high as 95.45%, compared with 72.72% of traditional testing, further proving its superiority for complex infectious cases; its specificity (37.50%) was still inferior to conventional assays (62.50%), consistent with previous research conclusions (38).

Considering the complementary performance differences between different sequencing technologies, mNGS and targeted next-generation sequencing (tNGS) can form a strategic combination in clinical practice. tNGS features high sensitivity and low cost, which is suitable for rapid screening of common infectious pathogens. In contrast, mNGS with full-spectrum unbiased detection capacity acts as a final diagnostic guarantee for critical complex cases when conventional testing and tNGS fail to identify pathogens (39).

## 3 Four core cognitive revolutions brought by mNGS: reconstructing the pathogen spectrum map of severe pneumonia in children

mNGS breaks through the cognitive limitations of traditional pathogen detection, constructing a more detailed and complete pathogen spectrum map and thoroughly renewing the understanding of pathogenesis of severe pneumonia in children. From the clinical perspective, this technology brings four paradigm shifts that reshape the cognition of childhood pulmonary infectious diseases.

### 3.1 Viruses are core pathogens rather than secondary accidental infection

In the past, clinicians only paid close attention to influenza virus and respiratory syncytial virus (RSV), while ignoring the pathogenic role of other respiratory viruses. However, multiple mNGS-based studies have fully proved that the pathogen spectrum of severe pneumonia in children is highly diverse, and viruses rank first among all detected pathogens (26, 37).

Besides RSV and influenza virus, adenovirus, rhinovirus and human metapneumovirus have been confirmed as independent or major causative agents of children's pneumonia, which were previously regarded as low-virulence rare viruses (29, 35, 37). For critically ill or immunocompromised children, reactivation of herpesviruses including Epstein–Barr virus (EBV) and cytomegalovirus (CMV) is closely correlated with disease deterioration, further highlighting the central pathogenic status of viruses in children's pneumonia (35, 36, 40, 41).

### 3.2 Rare and emerging pathogens are frequently detected in clinical practice

Benefiting from its unbiased full-spectrum detection characteristic, mNGS becomes an effective tool to capture previously undetectable pathogenic microbes. It greatly improves the detection rate of *Pneumocystis jirovecii*, making it the preferred testing method for immunocompromised children without HIV infection (36, 37, 41). This technology can detect 23S rRNA resistance sites of *Mycoplasma pneumoniae*, avoiding time-consuming culture procedures for atypical pathogens such as *Legionella* and *Chlamydia*. Meanwhile, mNGS supports rapid and accurate identification of smear-negative *Mycobacterium tuberculosis*, invasive fungi including *Aspergillus* and Cryptococcus, so as to guide timely targeted intervention (30, 42, 43).

### 3.3 Mixed infection is the clinical norm rather than rare accidental condition

Traditional clinical cognition holds that pneumonia is induced by a single pathogen, but massive mNGS test results overturn this view. Multiple cohort studies show that mixed infections account for 40%–70% of all mNGS-positive cases of severe pneumonia in children (26, 28, 29, 34).

A retrospective study including 55 critically ill children with severe pneumonia in PICU reported that the incidence of mixed infection reached 80% (44/55), among which bacterial-viral co-infection was the most common pattern. Except for regular virus-bacteria mixed infection, complex multi-pathogen co-infection such as virus-virus and bacteria-fungi combination is also widely found in children with severe pneumonia (30, 36, 44, 45). Age significantly affects the composition of mixed infection: RSV combined with *Streptococcus pneumoniae* is the dominant co-infection pattern in infants younger than 12 months old.

Accurate identification of mixed infections carries vital clinical significance. It explains why empirical single-pathogen targeted antibiotics often achieve poor curative effect for some children, and reminds clinicians to prioritize mixed infection in differential diagnosis and formulate combined individualized initial treatment regimens. It should be noted that unified diagnostic standards for mixed infection have not been formed in academia, which restricts quantitative evaluation of mNGS's diagnostic value for co-infections. Consistent judgment criteria need to be established in follow-up research to clarify its clinical application value.

### 3.4 Redefining the boundary between microbial colonization and invasive infection

The ultra-high sensitivity of mNGS enables simultaneous detection of dozens of microorganisms in one specimen, making it difficult for clinicians to distinguish true pathogens from colonizing or contaminating flora in complex sequencing data, which becomes a major obstacle for clinical interpretation (9). Recent researches propose multi-dimensional comprehensive interpretation strategies to solve this difficulty.

First, semi-quantitative analysis based on pathogen relative abundance and unique sequencing reads can assist judgment: microbial species with extremely high abundance are more likely to be real pathogenic agents (47). Second, researchers can build a background flora database combining respiratory microbiome characteristics of healthy populations and laboratory environmental microbial libraries to automatically filter common colonizers and laboratory contaminants. Most importantly, all sequencing results must be interpreted combined with children's clinical manifestations, immune status and imaging findings. When necessary, dynamic changes of pathogen abundance before and after treatment can be tracked to confirm microbial virulence. Existing research has verified the correlation between mNGS-detected microbes and host clinical characteristics, proving the feasibility of this comprehensive interpretation strategy (46, 47).

## 4 Clinical transformation of mNGS: from theoretical advantages to precise treatment of severe pneumonia in children

The cognitive revolution of pathogen spectrum driven by mNGS promotes the treatment model of severe pneumonia in children to shift from blind empirical medication to standardized precision therapy, and these theoretical advantages have been fully transformed into tangible clinical benefits.

In etiological diagnosis of severe pneumonia in children, mNGS owns three core clinical strengths. First, it efficiently identifies all types of mixed infections including virus-virus, virus-bacteria and bacteria-fungi co-infection, which are prevalent among critically ill children. Second, it outperforms traditional testing in capturing fastidious, rare, emerging and non-culturable pathogens that cannot be detected by conventional methods. Third, mNGS synchronously detects pathogenic microorganisms and antimicrobial resistance (AMR) genes, providing molecular evidence for accurate antibiotic selection, de-escalation therapy and rational control of antimicrobial consumption (19).

Combined, these strengths significantly improve diagnostic accuracy, optimize treatment schemes and ameliorate clinical prognosis of children with severe pneumonia, showing outstanding value in guiding targeted therapy, resolving complex critical cases and exploring prognostic management strategies.

The most prominent clinical value of mNGS lies in guiding targeted intervention and standardizing rational antibiotic use. Multiple clinical trials confirm that sequencing results lead to adjustment of antibiotic regimens in more than half of children (26, 48). Different from culture-dependent resistance testing, mNGS can directly capture AMR gene information from BALF or blood specimens without microbial isolation, providing molecular-level basis for personalized precision treatment (34, 48, 49).

By sequencing all nucleic acids in clinical specimens, mNGS simultaneously detects various pathogens and corresponding resistance genes. It can identify unculturable pathogens and their carrying drug-resistant determinants that cannot be captured by traditional testing, guiding the formulation of individualized antimicrobial plans for pneumonia and bloodstream infection (30, 50). This technical advantage translates into two clear clinical benefits.

On one hand, mNGS can detect drug-resistant bacteria, fungi or *Mycobacterium tuberculosis* missed by conventional testing, allowing clinicians to switch to effective targeted drugs rapidly, avoid disease deterioration caused by inappropriate medication and shorten children's hospital stay. On the other hand, if mNGS only detects viruses or fully susceptible bacteria without virulent co-pathogens in children receiving empirical broad-spectrum antibiotics, clinicians can safely downgrade broad-spectrum antibiotics to narrow-spectrum agents or completely stop antimicrobial treatment. This de-escalation strategy based on full-spectrum pathogen and resistance screening reduces adverse drug reactions, suppresses the generation of bacterial resistance and cuts overall treatment costs (51, 52).

For example, a child with fever and pulmonary infiltration receives empirical combined therapy of azithromycin and third-generation cephalosporins. If mNGS detects high adenovirus load without any pathogenic bacteria or resistance genes, clinicians can completely withdraw antibiotics and shift to symptomatic supportive care. This typical clinical scenario fully reflects how mNGS promotes the transformation from blind broad-spectrum empirical treatment to evidence-based individualized therapy for children with severe pneumonia.

For critically ill children with negative results of routine testing and unidentified etiology, mNGS provides key support for resolving difficult diagnostic cases and shortening the time to definite diagnosis (53). Its 24–48 h rapid reporting cycle meets the urgent clinical demands of PICU. In clinical practice, some children show recurrent high fever and poor response to multiple drugs with negative blood culture, sputum culture and targeted PCR results (17). Under such circumstances, mNGS often detects rare actinomycetes or endemic fungi, achieving a diagnostic breakthrough, greatly shortening the trial-and-error diagnosis cycle and reducing total hospital stay (43).

Additionally, mNGS has broad application prospects in disease risk stratification and personalized prognostic intervention. Beyond pathogen identification, it can characterize the overall composition of lung microbiome. Cutting-edge researches are exploring the predictive value of pathogen spectrum and microbial community structure for disease severity, disease course and long-term prognosis.

Relevant data prove that specific virus-bacteria co-infection modes correlate with prolonged PICU admission and higher mechanical ventilation rate (54). Based on microbial fingerprints generated by mNGS, researchers have put forward potential individualized intervention directions including risk stratification and targeted lung microbiome regulation, although related exploration is still in the initial stage. Existing clinical trials also confirm that early comprehensive pathogen identification through mNGS helps dynamically adjust antimicrobial regimens, improve clinical outcomes and potentially reduce mortality (31, 55).

## 5 Current challenges and future development directions of mNGS technology

mNGS greatly improves the detection efficiency of pathogenic microbes in severe pneumonia in children, lifting the overall pathogen positive rate from less than 50% of traditional testing to over 85% (10). Nevertheless, multiple bottlenecks in technical operation, result interpretation and ethical supervision restrict its full-scale clinical promotion, while clear development paths have been proposed to overcome these limitations.

### 5.1 Existing restrictions of mNGS

First, high single-test cost (several thousand RMB per specimen) limits wide application in resource-limited medical institutions and brings economic pressure to children's families (46). Its cost-effectiveness remains controversial: some studies report that mNGS reduces total medical expenditure by shortening hospital stay (21, 56, 57), while other researches show higher overall costs for mNGS-tested children due to selection bias toward critical cases.

Second, the experimental workflow of mNGS is far more complex than traditional testing, covering two core links: sequencing experiment (sample processing, nucleic acid extraction, library construction, on-machine sequencing) and bioinformatic analysis (27). BALF is the optimal specimen for lower respiratory tract infection diagnosis in children with severe pneumonia, as it directly reflects lesion pathogen spectrum and avoids upper respiratory flora interference (27, 34). However, BAL sampling relies on invasive fiberoptic bronchoscopy; non-standard sterile operation of environment, instruments or procedures will lead to specimen contamination. At present, unified industry standard operating procedures (SOPs) covering all links from sample delivery to bioinformatic analysis are absent, resulting in inconsistent test results across different laboratories (57).

Third, strict aseptic control is required in specimen collection, nucleic acid extraction, library construction and sequencing. Trace exogenous nucleic acid introduced during operation will produce detectable sequencing signals and trigger false-positive results. Exogenous pollutants originate from laboratory surfaces, experimental consumables, reagents and normal human commensal flora. Contaminated specimens interfere with bioinformatic analysis and distort final reports (46). The ultra-high detection sensitivity of mNGS puts forward extremely strict requirements for full-process aseptic quality control to avoid detection errors caused by trace pollutants (58).

Fourth, abundant host nucleic acid in BALF samples interferes with identification of low-abundance pathogens. Efficient removal of host background sequences remains a difficult technical problem restricting detection sensitivity improvement (59). Besides, mNGS may capture microbes recorded in databases but with unclear clinical pathogenicity, bringing huge judgment challenges to clinicians.

From ethical and regulatory perspectives, mNGS may accidentally detect latent viruses irrelevant to current acute illness but with potential long-term health risks, and the management and reporting of such information involve complicated ethical disputes. Furthermore, massive whole-genome sequencing data generated by mNGS contain host personal genetic information, so patient data privacy protection becomes an urgent regulatory problem to be solved.

### 5.2 Future development directions

Multiple technical routes have been proposed to solve the above limitations. In technical iteration, Oxford Nanopore nanopore sequencing technology represented by portable sequencing devices supports real-time ultra-long fragment sequencing, shortening pathogen identification time to several hours and realizing bedside rapid detection (60). Continuous optimization of portable sequencers improves the feasibility of mNGS application in resource-limited medical centers (61, 62). Ombelet et al. (63) put forward low-cost, simple-operation and low-maintenance testing equipment development goals, specifically promoting the popularization of mNGS in under-resourced regions.

To balance diagnostic accuracy and medical economic burden, clinical institutions should build a tiered diagnosis and treatment system for severe pneumonia in children, reasonably matching high-sensitivity mNGS and low-cost traditional culture, and optimize the whole diagnostic workflow.

Host transcriptome sequencing provides a new research perspective to distinguish colonization and invasive infection: researchers can combine mNGS pathogen data and host mRNA expression profiles to evaluate microbial virulence based on host immune response characteristics. Artificial intelligence (AI) technology also brings new breakthroughs for metagenomic data interpretation. Wani et al. (64)systematically summarized the synergistic value of metagenomics and AI in human health research, confirming that AI algorithms can efficiently process massive microbial genomic data and improve the accuracy and efficiency of pathogen identification and functional prediction.

Jiménez et al. (65) further studied the discovery and culture of prokaryotic microbes under the combination of metagenomics and artificial intelligence, highlighting the unique advantages of machine learning in identifying unknown microbial taxa and interpreting complex microbiome structures. Targeting pneumonia diagnosis, AI-assisted analysis has broad clinical prospects. Barbieri et al. (66) reviewed relevant researches and pointed out that AI can accurately predict hospitalization risk, complication occurrence and long-term prognosis of children with community-acquired pneumonia by integrating clinical and microbial data. Meanwhile, AI standardizes real-time clinical decision-making and reduces judgment deviation caused by subjective clinical experience. Ginsburg and McCollum (67) outlined the application prospects of artificial intelligence in integrated community-acquired pneumonia management, and noted that AI-based risk stratification systems facilitate rational allocation of medical resources and real-time dynamic patient monitoring.

In the future, artificial intelligence can integrate microbiome, host transcriptome and clinical phenotypic data to build intelligent evaluation models, realizing automatic accurate assessment of pathogen virulence and disease prognosis prediction. Large-sample, multi-center randomized controlled clinical trials are urgently needed in this field. Follow-up researches should clarify the clinical efficacy of mNGS for primary screening and secondary confirmatory diagnosis, quantify its influence on children's clinical outcomes and evaluate its long-term medical economic value, so as to provide high-level evidence for standardized clinical popularization.

The standardized clinical diagnosis and treatment system for severe pneumonia in children should incorporate mNGS into routine diagnostic procedures and build a closed-loop precision diagnostic model. Hospitals are recommended to establish multi-disciplinary interpretation teams (MDT) composed of pediatricians, professional microbiologists, bioinformatic analysts and clinical pharmacists. Through joint multi-disciplinary consultation, the team breaks through the single-discipline limitation of result judgment and completes rigorous evaluation of mNGS sequencing data.

Combined with children's specific clinical symptoms, physical signs, inflammatory markers, imaging manifestations and other conventional auxiliary test results, MDT teams eliminate the interference of colonizing and contaminating flora, accurately confirm true causative pathogens and formulate individualized diagnosis and treatment schemes matching children's actual conditions. This model maximizes the clinical application value of mNGS and significantly improves diagnostic efficiency, therapeutic effect and long-term prognosis of children with severe pneumonia.

Specific optimization schemes can be carried out at key detection links to improve overall testing efficiency. During nucleic acid extraction, laboratories can adopt special anti-pollution kits for specimen pretreatment, which effectively remove interfering impurities and inhibitors in samples, improve the enrichment degree, purity and recovery efficiency of target pathogen nucleic acid and reduce cross-contamination between specimens. Ensuring high-quality nucleic acid templates at the source lays a solid foundation for subsequent library construction, on-machine sequencing and bioinformatic analysis, guaranteeing the authenticity and reliability of final sequencing reports.

## 6 Conclusion

Benefiting from unparalleled full-spectrum detection depth and breadth, mNGS technology greatly expands and renews human cognition of the pathogen spectrum of severe pneumonia in children. It completely breaks through the inherent defects of traditional pathogen diagnosis relying on fixed detection targets and blind screening, opening a new era of full-coverage, high-precision precise pathogen identification.

mNGS accurately captures viruses long ignored by conventional testing, various emerging rare pathogens and highly prevalent mixed infection patterns in clinical practice. It provides core technical support for resolving complicated and critical diagnostic and therapeutic dilemmas of severe pneumonia in children, effectively promotes rational standardized use of antibiotics and implementation of individualized treatment plans, and lays a solid foundation for optimizing diagnosis and treatment strategies of children's infectious respiratory diseases.

Although mNGS still faces multiple challenges including high testing cost, absence of unified standardized operating specifications and difficulty of accurate clinical result interpretation, it has become an indispensable core component of the precision medicine system for severe pneumonia in children. With continuous technical iteration and optimization of clinical interpretation standards, mNGS and its derivative technologies will deepen human understanding of children's infectious diseases, continuously improve the diagnosis and treatment level of severe pneumonia, protect children's respiratory health and reduce childhood pneumonia mortality, bringing far-reaching clinical and public health benefits.
